# Natural variation in *HvAT10* underlies grain cell wall-esterified phenolic acid content in cultivated barley

**DOI:** 10.3389/fpls.2023.1095862

**Published:** 2023-05-10

**Authors:** Kelly Houston, Amy Learmonth, Ali Saleh Hassan, Jelle Lahnstein, Mark Looseley, Alan Little, Robbie Waugh, Rachel A. Burton, Claire Halpin

**Affiliations:** ^1^ Cell and Molecular Sciences, The James Hutton Institute, Scotland, United Kingdom; ^2^ Division of Plant Sciences, School of Life Sciences, University of Dundee at The James Hutton Institute, Scotland, United Kingdom; ^3^ School of Agriculture, Food and Wine, University of Adelaide, Urrbrae, SA, Australia

**Keywords:** barley, p-coumaric acid, ferulic acid, BAHD, HvAT10, grain, cell wall, malting

## Abstract

The phenolic acids, ferulic acid and *p*-coumaric acid, are components of plant cell walls in grasses, including many of our major food crops. They have important health-promoting properties in grain, and influence the digestibility of biomass for industrial processing and livestock feed. Both phenolic acids are assumed to be critical to cell wall integrity and ferulic acid, at least, is important for cross-linking cell wall components, but the role of *p*-coumaric acid is unclear. Here we identify alleles of a BAHD *p-*coumaroyl arabinoxylan transferase, *HvAT10*, as responsible for the natural variation in cell wall-esterified phenolic acids in whole grain within a cultivated two-row spring barley panel. We show that *HvAT10* is rendered non-functional by a premature stop codon mutation in half of the genotypes in our mapping panel. This results in a dramatic reduction in grain cell wall-esterifed *p*-coumaric acid, a moderate rise in ferulic acid, and a clear increase in the ferulic acid to *p*-coumaric acid ratio. The mutation is virtually absent in wild and landrace germplasm suggesting an important function for grain arabinoxylan *p*-coumaroylation pre-domestication that is dispensable in modern agriculture. Intriguingly, we detected detrimental impacts of the mutated locus on grain quality traits where it was associated with smaller grain and poorer malting properties. *HvAT10* could be a focus for improving grain quality for malting or phenolic acid content in wholegrain foods.

## 1 Introduction

Phenolic acids in the cell walls of cereals limit digestibility when grain or biomass is used for animal feed or processed to biofuels and chemicals. They are also important dietary antioxidant, anti-inflammatory and anti-carcinogenic compounds and contribute to beer flavour and aroma. The hydroxycinnamates, *p*-coumarate and ferulate (*p*CA and FA respectively), are the major phenolic acids in grasses. Both occur as decorations ester-linked to cell wall arabinoxylan and that can be released by alkali. Lignin also has pendant alkali-releasable esterified *p*CA decorations but FA in lignin can be incorporated directly into the growing polymer by ether linkages (Hatfield et al., 2017) that are not alkali-labile. FA in the cell wall can be involved in radical coupling to cross-link arabinoxylans to each other and to lignin, and it is this cross-linking that may impede digestibility (de Oliveira et al., 2015). There is some evidence for an involvement of *p*CA in cross-linking but, in general, the role of *p*CA in cell walls is less clear (Hatfield et al., 2017). In lignin, it may promote polymerisation of sinapyl alcohol monolignols and act as a termination unit (Ralph et al., 2004; Hatfield et al., 2017) but there are no tested hypotheses about its role when attached to arabinoxylans. However, both phenolic acids have been suggested to contribute to the mechanical strength and digestion-recalcitrance of grain hull cell walls, traits that might influence dormancy or survival, the properties of grain during industrial processing, and the release of nutrients and phytochemicals during digestion. The health-promoting properties of the phenolic acids and other phenylpropanoids, including their antimicrobial, anti-inflammatory, antioxidant, anticancer activities, and their influence on gut health and microbial composition, are all areas of current study (Neelam and Sharma, 2020; Tiozon et al., 2022).

Some but not all of the genes involved in phenolic acid incorporation into grass cell walls have been identified. A specific clade of BAHD acyltransferases, the ‘Mitchell’ clade (Mitchell et al., 2007), is known to encode enzymes involved in *p*CA and FA addition to an unknown acceptor molecule prior to incorporation into cell wall polymers, although the exact activity of some clade members is still an open question. A complete phylogenetic analysis revealed 20 such genes in the sequenced genome of rice and named them acyltransferases (AT) AT1 to AT20 with only AT1 to AT10 being relatively highly expressed (Bartley et al., 2013). There is currently great interest and research activity into manipulating these Mitchell clade genes in transgenic plants to infer function and to manipulate phenolic acid content to improve the health-promoting properties or industrial uses of cereal crops. A specific motivation of many studies has been to improve digestibility of plant biomass for biofuel and chemical production in future biorefineries. Experimental strategies have ranged from using transgenic activation-tagged mutants ectopically overexpressing BAHD genes (Bartley et al., 2013), to BAHD RNAi suppression or CRISPR/Cas9 knockout (Marita et al., 2014; Petrik et al., 2014; Buanafina et al., 2016; de Souza et al., 2018; Mota et al., 2021; Möller et al., 2022) or overexpression in homologous or heterologous species (Buanafina et al., 2016; Karlen et al., 2016; Sibout et al., 2016; Fanelli et al., 2021; Tian et al., 2021). Results have not always been completely consistent making interpretation difficult, particularly when comparing transgenic experiments using distinct species and genes where enzyme specificity or levels and tissue-localization of gene suppression/overexpression may differ. We therefore pursued an alternative approach, determining the genetic loci underlying the natural variation in cell wall esterified phenolic acids in the grain of elite barley cultivars. This approach should not only help in identifying some of the major determinants of cell wall *p*CA and FA content in grain, but should also provide natural variants that can be exploited more immediately than transgenic approaches for breeding improved crops.

## 2 Results

### 2.1 GWAS of cell wall-esterified *p*CA and FA quantified from whole grains

**Figure 1:**
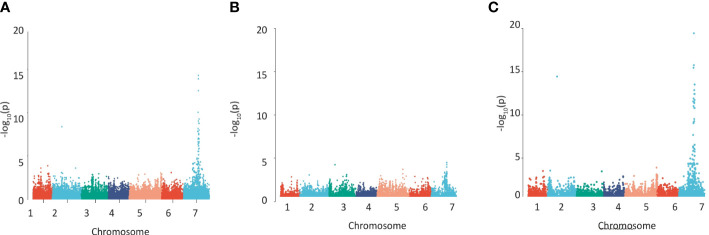
Detecting regions of the barley genome associated with grain phenolic acid content using a collection of 211 spring 2-row barleys. Manhattan plots of the GWAS of the phenolic acid content of wholegrain 2-row spring barley indicating regions of the genome associated with grain **(A)** *p*-coumaric acid, **(B)** ferulic acid content, **(C)** using the a ratio of these two phenolic acids calculated by log[FA:p-Coumaric acid]. The –Log 10 (P-value) is shown on the Y axis, and the X axis shows the 7 barley chromosomes. The FDR threshold = −log 10(P)=6.02, plots use numerical order of markers on the physical map.

We quantified cell wall-esterified *p*CA and FA in the wholegrain of a replicated GWAS panel of 211 elite 2-row spring barley cultivars grown in a field polytunnel. We observed a 6-fold variation for esterified *p*CA (54 µg/g - 327 µg/g) and a greater than 2-fold variation in esterified FA (277 µg/g -748 µg/g) (**Supplementary Figures 1A, B**, **Supplementary Datas 1**, **2**). A GWAS of this data using 43,834 SNP markers identified a single highly significant association peak for grain esterified *p*CA on chromosome 7H (**Figure 1A**, **Supplementary Data 3**) and a co-locating peak for FA just below statistical significance (**Figure 1B**, **Supplementary Data 3**). The peak region above the adjusted false discovery rate (FDR) threshold for *p*CA spanned a 114.4 MB region from 461,023,389bp – 575,489,630bp. The peak for FA showed linkage between markers for a 59.0 MB region from 518,604,829bp – 577,609,887bp.

Given the closeness of FA and *p*CA on the phenylpropanoid pathway we also conducted a GWAS using FA:*p*CA concentration ratios which provides internal data normalisation and reduces the inherent variability associated with measuring the single compounds (Petersen et al., 2012). Mapping FA:*p*CA ratios as log[FA:*p*CA] values increased both the strength and significance of association with the locus (i.e. the -log10(p) for *p*CA is 13.9 and for FA is 3.9 but for FA:*p*CA it rises to 19.4; **Figure 1C**, **Supplementary Figure 2C**, **Supplementary Data 3**). This increase in the significance of the association when the phenolic acid ratios are used instead of the single compound values confirms a level of dependency between esterified FA and esterified *p*CA concentrations. GWAS on similar data from a semi-independent set of 128 greenhouse-grown barley genotypes identified the same associations (**Supplementary Figures 2A–C**, **Supplementary Data 3**).

### 2.2 Identification of candidate genes on chromosome 7H

**Figure 2:**
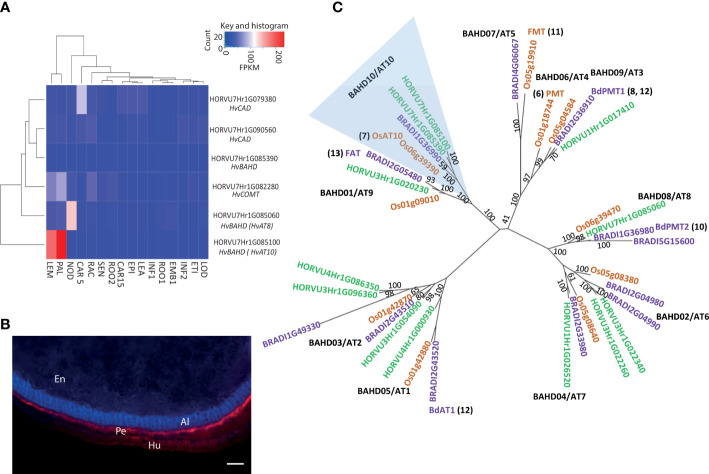
Putative candidates contributing to variation in grain *p*-coumaric (*p*CA) and ferulic acid (FA) content from GWAS. **(A)** Expression pattern for candidate genes under GWAS peak on 7H for grain *p-*coumaric and ferulic acid content in 16 different tissues/developmental stages. Tissues are (where DPA is ‘days post anthesis’ and pa is ‘post anthesis’): LEA=Shoot (10cm seedlings), EPI=Epidermis (4 weeks), CAR15=Grain (15 DPA), ROO2=Root (4 week seedling), SEN=Senescing leaf (2 months), LOD=Lodicule (6 weeks pa), ETI=Etiolated (10 day seedlings), INF1=Inflorescence (0.5cm), EMB=Embryo (germinating), ROO1=Root (10cm seedlings), INF2=Inflorescence (1-1.5cm), RAC=Rachis (5 weeks pa), CAR5=Grain (5 DPA), NOD=Tillers (3rd internode), PAL=Palea (6 weeks pa), LEM=Lemma (6 weeks pa). Values are FPKM and a scale bar is provided. This expression data is derived from the publicly available RNAseq dataset BARLEX, https://apex.ipk-gatersleben.de/apex/f?p=284:39. **(B)** Autofluorescence in whole grain sections revealing tissue structure. En: Endosperm, Al: Aleurone, Pe: Pericarp and Hu: Husk. Scale bar = 100μm **(C)** Phylogenetic tree of the *BAHD* acyltransferases. A maximum-likelihood tree of the translation alignment of the coding sequences of group A and B *BAHD* genes from barley, rice and *Brachypodium*. Bootstrap support for branches is provided. Horvu numbers represent the barley gene models in green, BRADI represents *Brachypodium* in purple, and *Os* represents the rice genes in orange. The clade including LOC_OS06g39390 and HORVU7Hr1G085060 is highlighted in blue, *OsAT10* is indicated in green and the closest barley orthologue is marked in red. Where function of a gene model has been assigned the relevant reference is provided. Black text in bold indicates branch names, both BAHD and AT (as in de Souza et al., 2018).

The entire peak region above the adjusted false discovery rate (FDR) threshold for the log[FA:*p*CA] values spanned a 65.7MB segment of chromosome 7H, from 459,131,547bp to 524,825,783bp and this region contains 347 high-confidence gene models. We surveyed this region for genes involved in phenolic acid or cell wall biosynthesis. This revealed several candidates including two cinnamyl alcohol dehydrogenases (*CAD*s), a caffeate-O-methyltransferase (*HvCOMT1*; Daly et al., 2019) and three BAHD acyltransferases. Interrogation of an RNA-seq dataset for 16 barley tissues (Colmsee et al., 2015) revealed that five of these six candidates showed only moderate to low levels of expression across all surveyed tissues (**Figure 2A**). However, the *BAHD* gene HORVU7Hr1G085100 stood out as being highly expressed in the husk lemma and palea where 80% of grain *p*CA is found (Barron et al., 2017) (**Figures 2A, B**). We then consulted a database of variant calls from a barley RNA-seq dataset that included 118 of our GWAS genotypes (Rapazote-Flores et al., 2019). We observed no SNP variation in two of the candidate genes. Three had one SNP each; *COMT1* (HORVU7Hr1G082280) had a synonymous SNP, one *CAD* (HORVU7Hr1G079380) had a SNP in the 3’ UTR and one *BAHD* (HORVU7Hr1G085390) had a non-synonymous but rare SNP. None of these SNPs appeared likely to impair gene function. However, the *BAHD* HORVU7Hr1G085100 had 3 SNPs including one causing a premature stop codon that would lead to loss of a third of the protein sequence. BLASTp of the predicted full-length HORVU7Hr1G085100 protein sequence revealed it was 79% identical to rice *OsAT10* (LOC_Os06g39390.1), a gene previously functionally characterised as a *p*-coumaroyl CoA arabinoxylan transferase (Bartley et al., 2013). Critically, ectopic overexpression of *OsAT10* in rice dramatically increases cell wall-esterified *p*CA levels in leaves while concomitantly reducing the levels of esterified FA (Bartley et al., 2013). A maximum likelihood phylogenetic tree of *BAHD* gene sequences confirmed HORVU7Hr1G085100 as the barley ortholog of rice *OsAT10*, i.e. *HvAT10* (**Figure 2C**). Another of our candidates, HORVU7Hr1G085390, is a possible *HvAT10* paralog but has negligible expression in the tissues surveyed (**Figure 2A**). The third *BAHD*, HORVU7Hr1G085060, is likely an AT8 (Bartley et al., 2013; de Souza et al., 2018). A gene recently suggested to act as a *p*-coumaroyl CoA arabinoxylan transferase in vegetative tissues in *Setaria viridis* (Mota et al., 2021) has a barley orthologue on chromosome 4H (AT1 clade, **Figure 2C**), remote from our 7H grain *p*CA/FA locus.

### 2.3 Discovery of causal mutation – a premature stop codon in *HvAT10*

**Figure 3:**
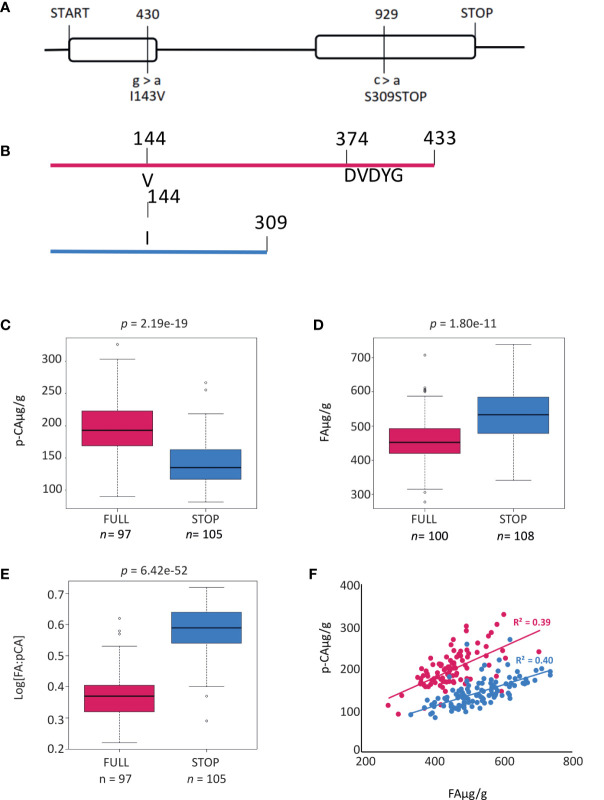
Gene and protein models for *HvAT10*. **(A)** Gene model for *HvAT10* including location, and effect of SNPs detected from resequencing this gene in the 211 barley cultivars which have been assayed for *p*-coumaric and ferulic acid. The numbering above the gene model represent locations in the CDS which vary between these cultivars. The SNP, and the resulting change in the particular amino acid are indicated underneath the gene model. The full length of the gene is 2117bp (with a CDS of 1302bp) which translates to a protein of 435 amino acids as indicated. Protein model for translation of HvAT10. **(B)** a Full length protein and **(C)** when the premature stop codon is present this results in a truncated protein. Box plots demonstrate the effect of the SNP at 929bp within *HvAT10* where the grain of the 211 barley cultivars were quantified for **(D)** *p*-coumaric acid levels and **(E)** ferulic acid levels. **(F)** Correlation between *p*CA and FA content based on *HvAT10* allele using 211 lines. The allele which results in full length version of HvAT10 are in pink, and the allele leading to a premature stop codon are coloured blue.

To more accurately document polymorphisms in *HvAT10*, we PCR-sequenced the gene from 52 genotypes of the GWAS panel (**Supplementary Data 1**). Two nonsynonymous SNPs, one in each of *HvAT10*’s two exons (**Figure 3A**), were in complete linkage disequilibrium across the 52 lines. A G/A SNP at 430bp translates to either a valine or isoleucine, substituting one non-polar, neutral amino acid for another, so unlikely to affect function. By contrast, a C/A SNP at 929bp produces either serine in the full length protein, or a premature stop codon that truncates the protein by 124 amino acids, removing the BAHD family conserved DFGWG motif (DVDYG in barley and other grasses) thought to be essential for catalysis (Morales-Quintana et al., 2015) (**Figure 3B**). The *at10^STOP^* mutation is therefore predicted to knock-out gene function. We designed a diagnostic Kompetitive Allele Specific PCR (KASP) assay to distinguish the wildtype and mutant *HvAT10* alleles and genotyped all 212 cultivars in our GWAS population (**Supplementary Table 1**). Consistent with the hypothesis that *at10^STOP^* is the causal variant mutation underlying the FA:*p*CA ratio GWAS peak, no SNP scored higher than the KASP diagnostic when included in the GWAS although one, JHI-Hv50k-2016-488774, in complete LD, scored equally highly. *HvAT10* had a minor allele frequency of 0.48 and appears to significantly influence levels of both *p*CA (*p*=4.30e-19) and FA in grain (p=1.80e-11). The median grain esterified-*p*CA content for mutant *at10^STOP^* genotypes was 28% lower than it was for genotypes with the wildtype allele (**Figure 3C**), while median grain esterified-FA content was 14% higher for mutant *at10^STOP^* genotypes than it was for wildtype genotypes (**Figure 3D**). Comparing the median log[FA:*p*CA] for *at10^STOP^* cultivars (0.58) to the wildtype cultivar group (0.37) showed an even higher significant difference between the groups (*p*= 7.56e-50) (**Figure 3E**, **Supplementary Figure 3**).

### 2.4 Consequences of *HvAT10* premature stop codon mutation on *p*CA and FA in whole grain

Splitting the entire population of genotypes into the two allele groups (mutant and wildtype), and then plotting grain esterified *p*CA against FA within each group, revealed positive correlations between *p*CA and FA content in both allele groups (**Figure 3F**). This suggests that although flux into phenolic acid biosynthesis may differ between cultivars, it co-ordinately affects both phenolic acids irrespective of *Hv*AT10 allele. The *at10^STOP^* genotypes as a group show approximately one-third less *p*CA than wildtype genotypes (compare blue and red lines, **Figure 3F**), reflecting a deficiency of *p*CA on arabinoxylan in cultivars that lack a functional AT10 *p*-coumaroyl CoA arabinoxylan transferase. Nevertheless, two-thirds of cell wall esterified *p*CA remains, likely because most *p*CA is associated with lignin (Ralph, 2010; Lapierre et al., 2018) through the action of other BAHD genes. The influence of *at10^STOP^* on FA is evidenced by considering the 27 cultivars with grain esterified FA above 600 µg/g; 23 of these have the *at10^STOP^* allele (extreme right hand section of **Figure 3F**; **Supplementary Data 1**).

### 2.5 *HvAT10* mutation is virtually absent in wild and landrace barley

Intrigued by the prevalence of the *at10^STOP^* mutation in 50% of our elite barley genepool we were curious about whether this had any ecological, evolutionary, or performance-related significance. To explore this, we PCR-sequenced a collection of 114 georeferenced barley landraces, including accessions from Europe, Africa, and Asia, and 76 wild barley (*Hordeum spontaneum*) genotypes (Russell et al., 2016) across the *at10^STOP^* polymorphism (**Supplementary Data 1**). We found the *at10^STOP^* mutation to be extremely rare, present in only three of 114 landraces and absent in all 76 wild genotypes (**Supplementary Figure 4A**, **Supplementary Data 1**). The three *at10^STOP^* landraces show a clear pattern of identity by descent, clustering in the same clade of the dendrogram (**Supplementary Figure 4A**). We interpret these data as suggesting strong selection against the premature stop codon in wild germplasm and that *at10^STOP^* was a post-domestication mutation that under cultivation has no pronounced negative effects on fitness.

Several possibilities could explain enrichment of the *at10^STOP^* mutation in the cultivated genepool. To explore this, we first calculated the genome wide F_ST_ (fixation index) by locus using two groups based on the *HvAT10* allele. HORVU7Hr1G084140 (a serine/threonine-protein kinase not expressed in the lemma or palea) also had an F_ST_ of 1.0, and three other genes had an F_ST_ above 0.875 (**Supplementary Figure 5A, B**, **Supplementary Data 4**). Based on their functional annotations and gene expression patterns (**Supplementary Data 4**, **Supplementary Figure 5C**) we observed no obvious reason for these to be under strong selection and responsible for enhancing the frequency of the *at10^STOP^* mutation *via* extended LD.

### 2.6 Mutant locus influences grain size and malting properties

Next, due to the exclusive expression of *HvAT10* in the lemma and palea, we measured a series of grain morphometric traits across our panel. We found that, on average, grain from the *at10^STOP^* genotypes had significantly reduced grain width compared to cultivars with the wildtype allele (**Table 1**). While wildtype cultivars had an average grain width of 3.97mm, mutant *at10^STOP^* cultivars had a narrower width of 3.92mm and this difference was very highly significant (p=0.0009) suggesting a potential role for arabinoxylan-esterified phenolic acids in determining grain shape.

**Table 1:** T-test results for comparisons between *HvAT10* alleles.

	Grain Area	Grain Length (mm)	Grain Width (mm)	Hot water extract l°/kg	Germinative energy 4ml %	Fermentable extract	Diastatic power (loB)	Wort viscosity (mPa/s)	Friability %
***at10*** *^STOP^*	27,67	9,02	3,92	309,44	97,34	70,72	100,77	1,50	87,97
SD	1,48	0,37	0,10	3,39	0,16	0,38	14,18	0,04	5,23
n=	107	107	107	69	56	51	59	56	59
**WT**	28,16	9,09	3,97	311,23	97,40	70,88	106,07	1,48	89,95
SD	1,46	0,38	0,10	2,61	0,15	0,36	12,67	0,02	2,93
n=	96	96	96	84	74	72	75	73	73
**p value**	0,0214*	0,152	0,0009***	0,0005***	0,0206*	0,0203*	0,0282*	0,0009***	0,0123*

For grain area, length and width data are available in **Supplementary Data 1** and analysis was carried out using BLUPS derived from 2 – years’ worth of samples. Data used for comparison of hot water extract, germinative energy, fermentable extract, diastatic power, wort viscosity and friability between HvAT10 alleles are published (Xu et al., 2018). SD, standard deviation; n, number of cultivars. *P ≤ 0.05; **P ≤ 0.01; ***P ≤ 0.001.

Prompted by these observations and the prevalence of registered UK barley varieties in our panel, we then explored grain parameters recorded in an extensive historical dataset from the UK’s National and Recommended Lists trials 1988-2016 (Looseley et al., 2020). Different grain quality phenotypes were available for up to 107 of our cultivars. Group comparisons of wildtype and *at10^STOP^* genotypes revealed surprising differences for hot water extract, diastatic power, germinative energy in 4ml, and wort viscosity (**Table 1**). In all cases, the group of *at10^STOP^* cultivars had poorer quality, offering no evidence of positive selection during breeding. The variation associated with the *HvAT10* locus is however highly significant and of potential interest for optimising grain quality traits (**Table 1**).

### 2.7 Pedigree and prevalence of the HvAt10 mutation in cultivated germplasm

Finally, to understand more about the origin of the *at10^STOP^* mutation in elite germplasm, we investigated its occurrence in the pedigree of our GWAS population. The earliest cultivar with the *at10^STOP^* mutation is the cultivar Kenia (a cross between the Swedish landrace Gull and the Danish landrace Binder) released in 1931 and subsequently introduced into north-west European breeding programmes. Despite smaller grain and slightly poorer malting properties compared to its contemporary UK varieties (i.e. phenotypes consistent with our analyses of *at10^STOP^* mutation cultivars as a group), it established a long-standing position as a parent for further crop improvement due to its short stiff straw, earliness and high yield (Bell, 1951). Several decades later, *at10^STOP^* mutation-containing derivatives of Kenia, such as cv. Delta (National list 1959), were still being used as parents in our pedigree chart. We conclude that the continued prevalence of Kenia-derived germplasm may go some way to explaining the frequency of the *at10^STOP^* mutant allele in our population.

## 3 Discussion

The phenolic acids FA and *p*CA in the cell walls in grasses are assumed to be critical to cell wall integrity. FA, at least, is important for cross-linking cell wall components, but the function of *p*CA is unknown. Here, we show that the natural variation for *p*CA and FA in cultivated barley grain is due to a very common knock-out mutation in a gene that links *p*CA to cell wall arabinoxylan precursors. The mutation is associated with smaller grain and poorer malting properties. Surprisingly, the mutation is completely absent in wild barley, suggesting an important function for grain arabinoxylan p-coumaroylation in the wild that is dispensable in modern agriculture.

The *HvAt10* gene that underlies this variation is part of the ‘Mitchell’ clade (Mitchell et al., 2007) and, in barley, its expression appears to be largely restricted to developing grain lemma and palea (**Figure 2A**). In rice and sugarcane also, AT10s are predominantly or only expressed in inflorescences although Brachypodium and maize show broader expression profiles (Fanelli et al., 2021). Compatible with *HvAt10* expression in barley grain, the group of *at10^STOP^* cultivars that do not make a functional enzyme had distinct grain phenotypes i.e. narrower grain and reduced grain area compared to wildtype cultivars (**Table 1**). Xu et al. (2018) previously identified a QTL hotspot on chromosome 7H for traits including grain area, and grain width. The eight 9K iSelect markers defining their QTL can be positioned on the current barley physical map at 482-500MB on 7H, corresponding to the location of *HvAT10*. Wang et al. (2019) also identified a QTL for grain length:width, grain perimeter, and grain roundness at the same location. Bartley et al. (2013) noted changes in total seed mass per plant in the OsAt10-overexpressing rice mutant although Mota et al., 2021 noted no changes in BAHD RNAi-silenced lines of *Setaria viridis*. Collectively, these observations suggest a potential role for arabinoxylan-esterified phenolic acids in influencing grain development and shape, possibly through controlling stiffness and mechanical properties of grain husk. Differences in grain shape and husk mechanical properties could, in turn, explain the differences in malting properties between *at10^STOP^* and wildtype cultivars. We can only speculate as to why *HvAt10*’s influence on grain width or hull mechanical properties might have been more important to grain survival or dispersal in wild versus cultivated genotypes, although the better germinative energy score of genotypes with the functional *HvAt10* allele (**Table 1**) may provide some insight.

AT10s share a conserved role in the *p*CA decoration of hemicellulose in different grasses. Initial work on the rice *OsAT10* characterised an activation-tagged mutant that ectopically overexpressed the gene resulting a dramatic 300% increase in cell wall-esterified *p*CA in arabinoxylan in young leaves and concomitant 60% reduction in esterified FA (Bartley et al., 2013). Constitutive overexpression of the rice gene in switchgrass similarly increased *p*CA in green leaves while decreasing FA (Li et al., 2018), although overexpressing the same gene in sorghum increased xylan-bound *p*CA without reducing overall FA (Tian et al., 2021). Recently, CRISPR/Cas9 knock-out of O*sAT10* in rice resulted in mutant plants with approximately 40% decrease in cell wall esterified *p*CA and essentially devoid of *p*CA associated with arabinoxylan (no change in *p*CA associated with lignin) but with some compensatory increase in grain husk arabinoxylan FA (Möller et al., 2022). Overexpression of the orthologous sugarcane Sc*AT10* in maize resulted in up to 75% increase in total *p*CA content with the increase restricted to hemicellulose, while total FA was reduced (Fanelli et al., 2021).

While interpretation of experiments where genes are massively over- and miss-expressed can be complicated, it is notable that an inverse interaction between levels of *p*CA and FA was also seen in our elite barley populations of many distinct cultivars when genotypes with either the full length wildtype or *at10^STOP^* mutant alleles of *HvAt10* are compared. Collectively, genotypes with the *at10^STOP^* allele had a median *p*CA level 28% lower than those with the wildtype allele, but a median FA level 14% higher than wildtype genotypes (**Figure 3C**). This effect on FA might occur in several ways: *p*CA that cannot be esterified onto arabinoxylan could be methoxylated to produce FA thereby increasing FA pools for transfer onto arabinoxylan, or alternatively, *p*CA and FA may compete for transfer onto a shared acceptor (likely UDP-arabinose) before incorporation into arabinoxylan such that loss of *p*CA transfer by *HvAT10* leaves more free acceptor for FA transfer. Either mechanism could explain how *at10^STOP^* can indirectly increase grain cell wall esterified FA.

Since both phenolic acids are altered by *HvAt10* knock-out, we cannot be conclusive about which cell wall changes underly the grain shape and malting phenotypes – these could be due to changes in either *p*CA or FA content or to the ratio between them. It is notable in this context that FA dimerization in the cell wall has been proposed to increase crosslinks between arabinoxylan and lignin, thereby increasing wall rigidity and potentially limiting growth (Ishii, 1997; Hatfield et al., 2017).

Taken together, we conclude that the prevalence of the *at10^STOP^* mutant allele in our elite barley populations may simply be a straightforward genetic legacy of historical barley breeding and use of Kenia-derived germplasm. However, our data suggests that purging the *at10^STOP^* allele could assist the development of superior quality malting barley varieties. Conversely, much research has focussed on the beneficial bioactivity of FA in the diet, and use of the *at10^STOP^* allele could facilitate breeding for increased FA in wholegrain products.

## 4 Methods

### 4.1 Plant material and growth conditions

Two populations of 2-row spring type barley were used to carry out the GWAS. The first population includes 211 elite lines grown in a polytunnel under field conditions in Dundee, Scotland in 2011 (Oakey et al., 2013). For each line, 5 whole grains were ground to a fine powder using a ball mill (Mixer Mill MM400; Retsch Haan Germany) and stored in dry conditions until the HPLC analysis. The second population which was used for verification of the results of the analysis of the first subpopulation includes 128 elite lines grown in a glasshouse compartment in a mix of clay-loam and cocopeat (50:50 v/v) at daytime and night time temperatures of 22°C and 15°C respectively in The Plant Accelerator, Adelaide, Australia (Hassan et al., 2017). The collection of germplasm these populations are sampled from has minimum population structure while maintaining as much genetic diversity as possible. Mature grains were stored until phenolic acid content analysis.

### 4.2 Genotyping with SNP markers

All lines were genotyped using the 50K iSelect SNP genotyping platform described previously (Bayer et al., 2017). Prior to marker-trait association analysis, all markers with a minimum allele frequency of <5% and markers with missing data >5% were excluded from the analysis.

### 4.3 Phenotyping for cell wall-bound phenolic acids

A ~ 20 mg amount of wholegrain barley (i.e. including husk) was used per sample. *Trans*-ferulic and *trans*-*p*-coumaric acid standards were purchased from SIGMA Aldrich (Castle Hill NSW, Australia). Standards were prepared at 62.5 µm, 250 µm and 1000 µm by dissolving the appropriate amount of powder in 50% methanol. Extraction of cell wall esterified phenolic acids was carried out following a previously described method (Irakli et al., 2012) with the following modifications. Samples were washed twice with 500 µl 80% ethyl alcohol, with shaking for 10 minutes at room temperature to remove free phenolic acids. To release total cell wall esterified phenolic acids, alkaline treatment was carried out by adding 600 µl 2M NaOH to the pellet. Samples were incubated on a rotary rack under nitrogen for 20 h in the dark at room temperature. Samples were centrifuged at 15000 x g for 15 minutes at room temperature, after which the supernatant was collected, acidified by adding 110 µl concentrated HCL and extracted three times with 1 mL ethyl acetate. Following each extraction, samples were centrifuged at 5000 x g for 7 minutes and the organic solution was collected. Extracts were combined, evaporated to dryness in a rotary evaporator and dissolved in 100 µl of 50% methanol prior to injecting 40 µl into the HPLC column. For each sample two technical replicates were applied.

### 4.4 HPLC conditions

An Agilent Technologies 1260 Infinity HPLC equipped with a Diode Array detector was used. Samples were analysed on an Agilent Poroshell 120 SB-C18 3.0x100mm 2.7- micron column kept at 30 C˚. Eluents were A (0.5mM trifluoroacetic acid) and B (0.5mM trifluoroacetic acid, 40% methanol, 40% acetonitrile, 10% water). Starting conditions were 85% A and 15% B. Flow rate was 0. 7 mL/min. Eluting gradients were as follow; min 0-10: 15% to 55% B, min 11-12: column washed with 100% B, min 13 back to the starting condition (85% A and 15% B). Detection was carried out at 280 nm and spectral data was collected from 200 to 400 nm when required. Ferulic and *p*-coumaric acid peaks were identified by comparing retention times and spectra to their corresponding standards. The area under the peaks was quantified at 280 nm for *trans* forms.

### 4.5 GWAS analysis of grain alkaline extractable *p*CA and FA and FA:*p*CA ratio

Marker- trait association analysis was carried out using R 2.15.3 (www.R-project.org) and performed with a compressed mixed linear model (Zhang et al., 2010) implemented in the GAPIT R package. For phenotype values, the mean values of the barley wholegrain total alkaline extractable *trans*-ferulic and *trans*- *p*-coumaric acid (w/w) were used. To identify genes within intervals associated with our trait we used BARLEYMAP (Cantalapiedra et al., 2015). We also used the ratio of FA:*p*CA as a trait in our GWAS analysis. The ratio between the two compounds was log transformed i.e. log(FA:*p*CA) to provide a more normally distributed dataset. When using ratios in GWAS, a significant increase in the *p*-gain statistic (a comparison between the lowest -log10(p) values of the individual compounds and the -log10(p) value of the ratio; Petersen et al., 2012) indicates that ratios carry more information than the corresponding metabolite concentrations alone. A significant p-gain identifies a biologically meaningful association between the individual compounds. We used B/(2*α) to derive a critical value of 3.42x10^5^ for the FDR-adjusted p-gain, where α is the level of significance (0.05) and B the number of tested metabolite pairs (Petersen et al., 2012). Therefore, as we tested two traits our threshold was 2 x 10^1^ and our p-gain was above this threshold.

To identify local blocks of LD, facilitating a more precise delimitation of QTL regions Linkage disequilibrium (LD) was calculated across the genome between pairs of markers using a sliding window of 500 markers and a threshold of R^2^<0.2 using Tassel v 5. We anchored markers that passed FDR and represented initial borders of the QTL on 7H to the physical map and then expanded this region using local LD derived from genome wide LD analysis as described above. When the GWAS had not resulted in an association that passed the FDR we used the arbitrary threshold of -LOG10(P) to define the initial border. The SNP with the highest LOD score was used to represent the QTL. After identification and Sanger sequencing of the candidate gene *HvAT10* the GWAS was repeated including the allele present at the S309Stop as an additional marker. The physical map positions and gene model names used in this analysis are from Morex v1 (Mayer et al., 2012, Mascher et al., 2017).

### 4.6 Bioinformatics and gene identification

We used BARLEX (Colmsee et al., 2015) to identify gene models present with the QTL defined by our analysis and their expression profile based on RNAseq data in 16 different tissues/developmental stages.

### 4.7 Phylogenetic analysis of barley BAHD acyltransferases

Coding sequences of all BAHD acyltransferases with the PFAM domain PF02458 from rice, barley and *Brachypodium* were downloaded from the Ensembl Plants database (http://plants.ensembl.org/). Sequences were aligned using the MUSCLE alignment function available in the Geneious 9.1.4 (https://www.geneious.com). The translation alignment option was used. A neighbour-joining tree was produced from the alignment. Barley genes within group A and B clades were identified, realigned with their rice and *Brachypodium* orthologs and a maximum likelihood tree was produced from the translation alignment of the sequences. The following settings were applied: substitution model: General-Time-Reversible (GTR), branch support: bootstrap, number of bootstrap: 1000.

### 4.8 Resequencing and genotyping of *HvAT10* in the main and supplemental set

Aligning the translation of AK376450 to Os06g39390 allowed the identification of the putative genomic sequence of *HvAT10.* We designed four pairs of primers, details of sequences and reaction conditions are in **Supplementary Data 5**, to amplify the full length CDS. To facilitate quick and efficient genotyping of large numbers of cultivars we subsequently designed a KASP genotyping assay to a SNP at 430bp in *HvAT10* (**Supplementary Data 5**) (Semagn et al., 2014). Reactions were performed in an 8.1 µL reaction volume, with 3 µL H2O, 1 µL DNA (20ng/µl), 4 µL KASP genotyping master mix, and 0.11 µL of the KASP assay.

Box plots to demonstrate the contribution of the SNP at 436bp in *HvAT10* to variation in grain *p*CA and FA content were produced using R 2.15.3 (www.R-project.org). To test for identity by descent of the *HvAT10* allele within the set of accessions using for the GWAS a dendrogram was constructed using maximum likelihood using the genotypic data from the 9k-select array in MEGA7 with default settings except for including bootstrapping and visualised in FigTree (v.1.4.4) http://tree.bio.ed.ac.uk/software/figtree/.

### 4.9 Characterization of diversity of *HvAT10* in *Hordeum spontaneum* from the fertile crescent and barley landraces

DNA was extracted as described above from 76 *H. spontaneum* and 114 barley landraces from (Russell et al., 2016). The S309Stop SNP was PCR amplified and Sanger sequenced with primer pair 5 using conditions described above. A dendrogram was constructed using maximum likelihood using 4000 exome capture derived SNPs from Russell et al. (2016) in MEGA7 with default settings except for including bootstrapping and visualised in FigTree (v.1.4.4) http://tree.bio.ed.ac.uk/software/figtree/.

### 4.10 Genome wide F_ST_ analysis

The fixation index (FST) is a measure of genetic differentiation between groups of individuals. Genome wide FST was calculated by locus using GenAlEx 6.502 after dividing the accessions into two populations based on their HvAT10 allele using all informative 50K iSelect markers.

### 4.11 Phenotypic analysis of cultivars with wildtype vs *at10^STOP^* allele

We characterised mature grain morphology using from plants grown in a polytunnel under field conditions in Dundee, Scotland as described above, over two years (2010 and 2011). Grain area, width and length were quantified using the MARVIN Seed Analyzer (GTA Sensorik GmbH, 2013). BLUPs (Best Linear Unbiased Predictions) calculated from this data using R 2.15.3 (www.R-project.org) were used in subsequent comparisons between allelic groups.

## Data availability statement

The data presented in the study are deposited in the NCBI repository, accession numbers OQ320054 - OQ320323..

## Author contributions

RW, KH, RB, CH, ALi, designed experiments. KH, AH, JL, ALe, carried out experiments. KH, AH, ALe, ML, ALi, JL analyzed data. The manuscript was written by CH, KH, RW, RB, ALe, AH with contributions from all other authors. All authors contributed to the article and approved the submitted version.

## Supplementary material

Supplementary Figure 1 Phenolic acid content of wholegrain flour from 211 2-row spring barleys linea. **(A)** *p*CA and **(B)** ferulic acid content. Values represent the mean for FA and pCA expressed as w/w. Error bars represent standard deviation of the replicates.

Supplementary Figure 2 Manhattan plots of the GWAS of the phenolic acid content of wholegrain flour from 128 2-row spring barley lines indicating regions of the genome associated with grain phenolic acid content. Manhattan plots of the GWAS of the phenolic acid content of wholegrain 2-row spring barley indicating regions of the genome associated with grain a. *p*-coumaric acid, b. ferulic acid content and c. log[FA:p-Coumaric acid]. The –Log 10 (P-value) is shown on the Y axis, and the X axis shows the 7 barley chromosomes. FDR threshold = −log 10(P)=6.02, plots use numerical order of markers on the physical map.

Supplementary Figure 3 Distribution of ratio between two phenolic acids quantified in the grain of 211 spring 2 row barleys lines and used to carry out GWAS. The ratio was calculated as log[FA:p-Coumaric acid]. Accessions containing the allele which results in a full length version of *HvAT10* are in pink, and accessions containing the allele leading to a premature stop codon are coloured blue.

Supplementary Figure 4 Distribution of the *HvAT10* premature stop codon in *H. vulgare* landraces and cultivated barley lines. **(A)** A dendrogram of 114 *H. vulgare* landraces constructed using a selection of SNPs with a genome-wide distribution with maximum likelihood methods. **(B)** A dendrogram of cultivated barley germplasm using a selection of SNPs with a genome-wide distribution using maximum likelihood methods. Accessions containing the allele which results in full length version of HvAT10 are in pink, and accessions containing the allele leading to a premature stop codon are coloured blue.

Supplementary Figure 5 F_ST_ analysis based on *HvAT10*. **(A)** Plot displaying genome wide F_ST_ with F_ST_ index provided on the Y axis, an F_ST_ of 1 indicating a complete fixation of each allele within the two subpopulations determined by their allele of *HvAT10*. **(B)** Just F_ST_ of markers at 7H. Red box indicates location of the centromere. Two SNPs whose location overlap on this plot, including one in *HvAT10*, have an F_ST_ of 1.0. Note shape of peak appears different in **(A, B)** due to the difference in scale of the plots. **(C)** RNAseq data for genes with F_ST_>0.875 from 16 different tissues/developmental stages. Values are FPKM and a scale bar is provided. This expression data is derived from the publicly available RNAseq dataset BARLEX, https://apex.ipk-gatersleben.de/apex/f?p=284:39. Tissue abbreviations as in main paper text.

Supplementary Data Sheet 1 Phenolic acid and genetic data for all cultivars included in this study. *p*-coumaric and ferulic acid content, KASP data and NCBI number for those lines that where sequenced for *HvAT10* is included.

Supplementary Data Sheet 2 Summary of number of accessions used for each GWAS. A total of 211 accessions were included in the main dataset but data for both phenolic acids is not available for all lines, therefore the number of individuals included in different analysis varies. Includes number of accessions for the GWAS presented in the main and supplementary analysis for both individual trait and the ratio analysis. Number of individuals with each allele of *HvAT10* based on genotyping of A430G is also included.

Supplementary Data Sheet 3 Details of QTL identified on 7H for all analysis carried out. Physical location, LOD score, and 50k iSelect marker with the highest LOD score are provided. * indicates that analysis passed the FDR threshold of -log10(p)=6.1.

Supplementary Data Sheet 4 Gene models containing SNPs that have an F_ST_>0.875 when F_ST_ analysis carried out based on *HvAT10* allele. This table includes 50k iSelect marker name, the chromosome the marker is located on, gene model and annotation based on Morex v1 Gene Models (2016).

Supplementary Data Sheet 5 Details of primers and genotyping assays used in this study. This includes details of primers for Sanger sequencing and KASP genotyping assay sequence for *HvAT10*.
