# Supplementary figures and images for: Natural variation in HvAT10 underlies grain cell wall-esterified phenolic acid content in cultivated barley
Source: Front Plant Sci. 2023 May 10;14:1095862. doi: 10.3389/fpls.2023.1095862 (PMC10206312; doi:10.3389/fpls.2023.1095862)

a.

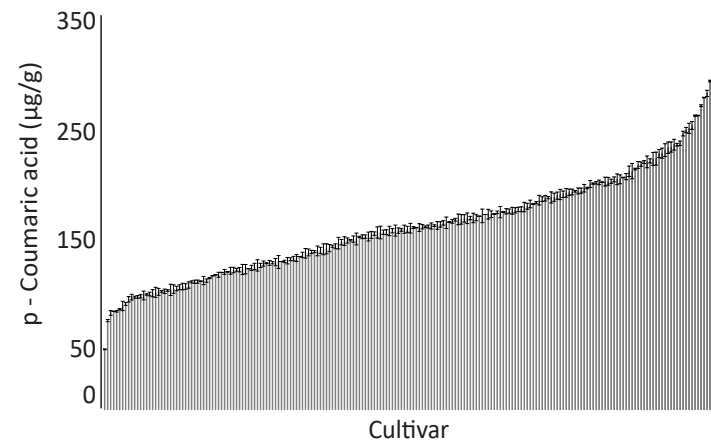

b.

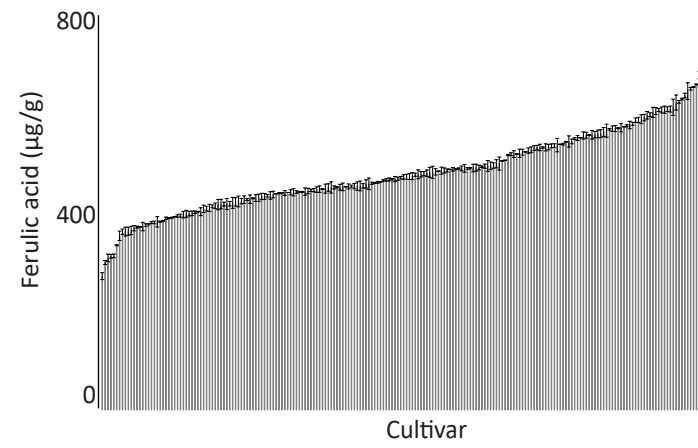

Supplement: Supplementary Figure 1 — Phenolic acid content of wholegrain flour from 211 2-row spring barleys linea. (A) pCA and (B) ferulic acid content. Values represent the mean for FA and pCA expressed as w/w. Error bars represent standard deviation of the replicates. [file DataSheet_1.pdf]

a.

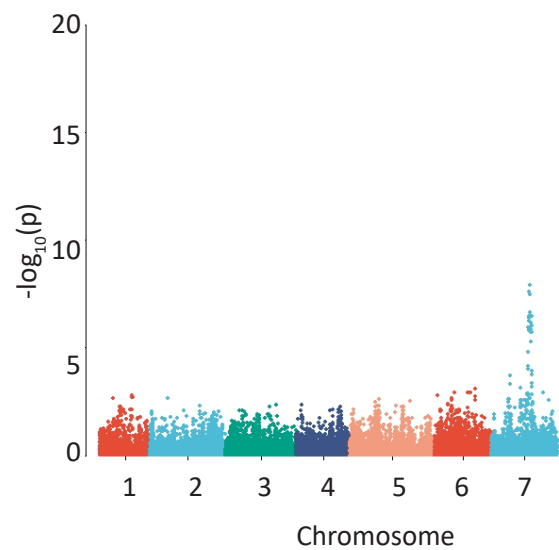

b.

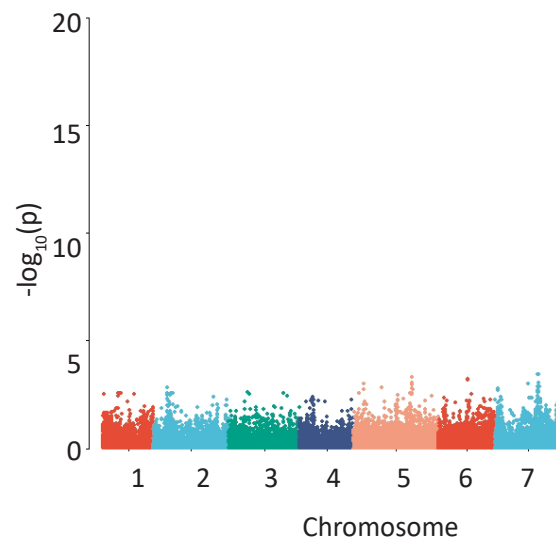

c.

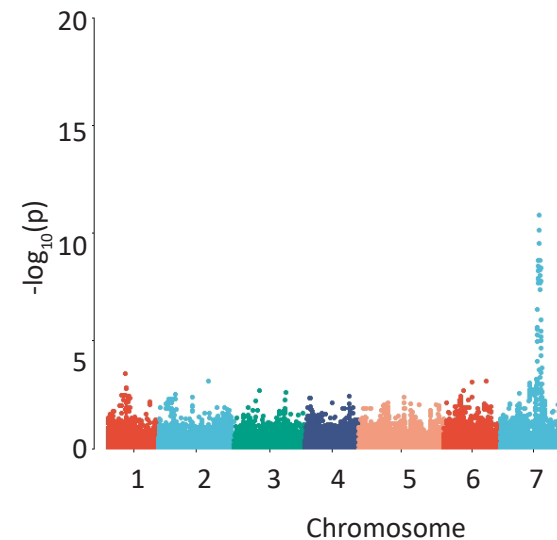

Supplement: Supplementary Figure 2 — Manhattan plots of the GWAS of the phenolic acid content of wholegrain flour from 128 2-row spring barley lines indicating regions of the genome associated with grain phenolic acid content. Manhattan plots of the GWAS of the phenolic acid content of wholegrain 2-row spring barley indicating regions of the genome associated with grain a. p-coumaric acid, b. ferulic acid content and c. log[FA:p-Coumaric acid]. The –Log 10 (P-value) is shown on the Y axis, and the X axis shows the 7 barley chromosomes. FDR threshold = −log 10(P)=6.02, plots use numerical order of markers on the physical map. [file DataSheet_2.pdf]

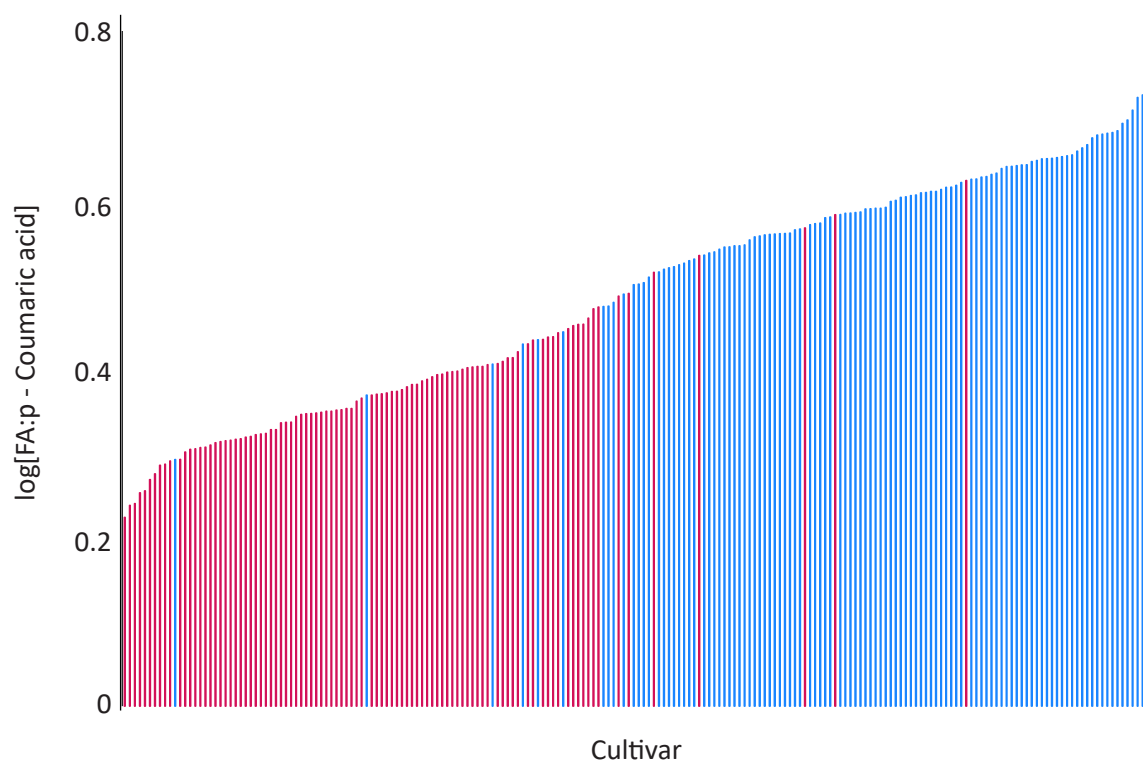

Supplement: Supplementary Figure 3 — Distribution of ratio between two phenolic acids quantified in the grain of 211 spring 2 row barleys lines and used to carry out GWAS. The ratio was calculated as log[FA:p-Coumaric acid]. Accessions containing the allele which results in a full length version of HvAT10 are in pink, and accessions containing the allele leading to a premature stop codon are coloured blue. [file DataSheet_3.pdf]

a.

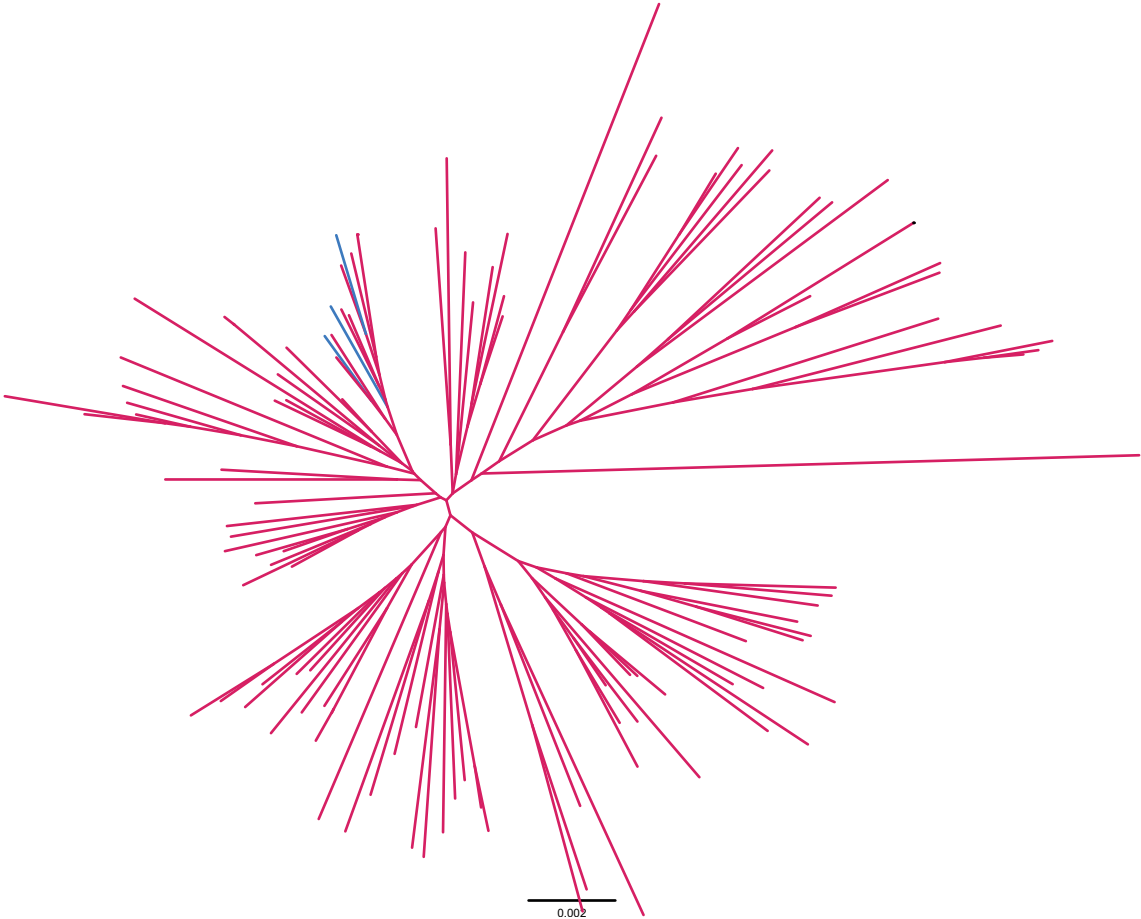

b.

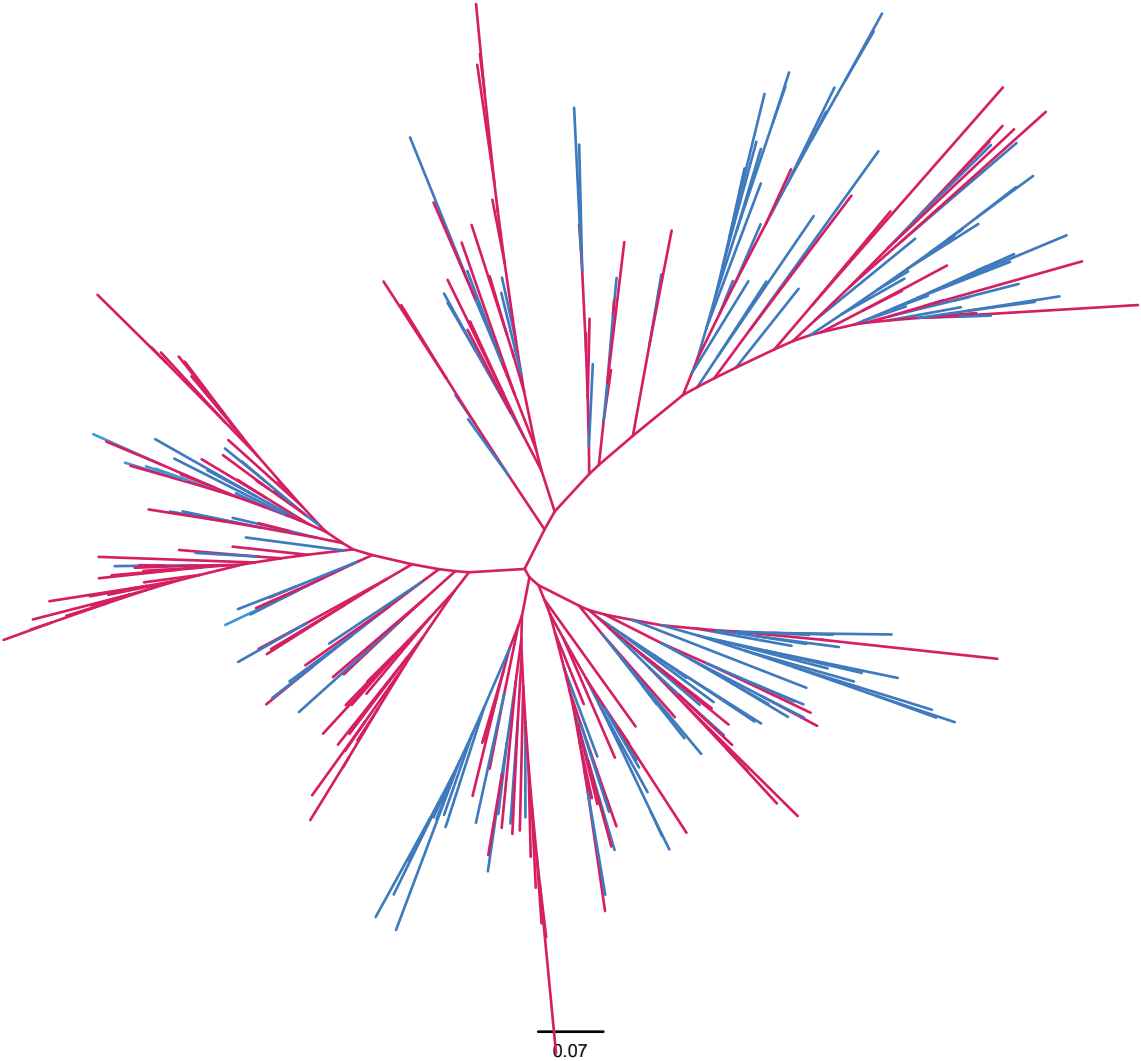

Supplement: Supplementary Figure 4 — Distribution of the HvAT10 premature stop codon in H. vulgare landraces and cultivated barley lines. (A) A dendrogram of 114 H. vulgare landraces constructed using a selection of SNPs with a genome-wide distribution with maximum likelihood methods. (B) A dendrogram of cultivated barley germplasm using a selection of SNPs with a genome-wide distribution using maximum likelihood methods. Accessions containing the allele which results in full length version of HvAT10 are in pink, and accessions containing the allele leading to a premature stop codon are coloured blue. [file DataSheet_4.pdf]

a.

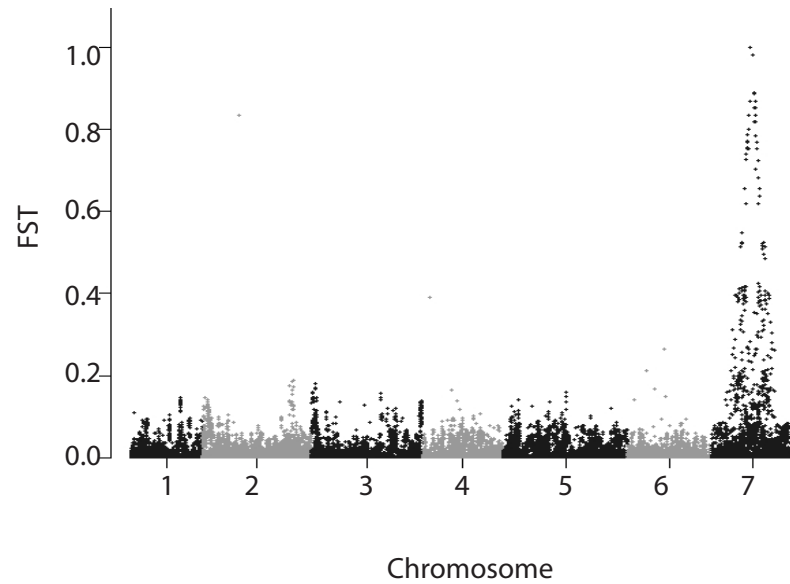

b.

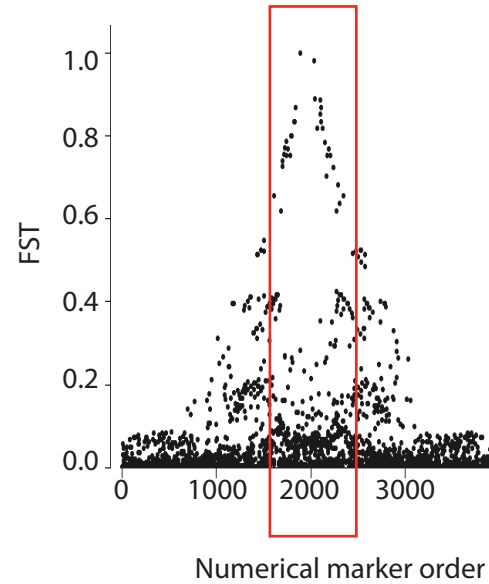

c.

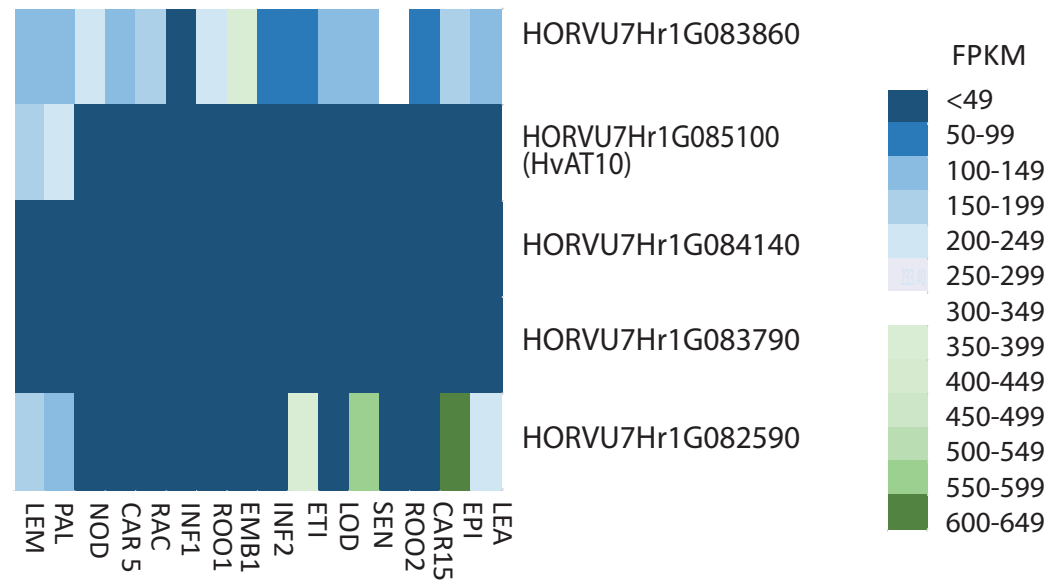

Supplement: Supplementary Figure 5 — FST analysis based on HvAT10. (A) Plot displaying genome wide FST with FST index provided on the Y axis, an FST of 1 indicating a complete fixation of each allele within the two subpopulations determined by their allele of HvAT10. (B) Just FST of markers at 7H. Red box indicates location of the centromere. Two SNPs whose location overlap on this plot, including one in HvAT10, have an FST of 1.0. Note shape of peak appears different in (A, B) due to the difference in scale of the plots. (C) RNAseq data for genes with FST>0.875 from 16 different tissues/developmental stages. Values are FPKM and a scale bar is provided. This expression data is derived from the publicly available RNAseq dataset BARLEX, https://apex.ipk-gatersleben.de/apex/f?p=284:39. Tissue abbreviations as in main paper text. [file DataSheet_5.pdf]
